# MRSA: A Challenge to Norwegian Nursing Home Personnel

**DOI:** 10.1155/2011/197683

**Published:** 2011-09-15

**Authors:** M. Thorstad, I. Sie, B. M. Andersen

**Affiliations:** ^1^Diakonova University College, 0166 Oslo, Norway; ^2^Department of Hospital Infections, Oslo University Hospital—Ullevål, 0407 Oslo, Norway

## Abstract

In Norway, methicillin-resistant *Staphylococcus aureus* (MRSA) is increasing in primary healthcare, associated with imported cases and outbreaks in long-term care. According to Norwegian national guidelines, MRSA-exposed healthcare workers (HCWs) and patients are tested. Carriage of MRSA leads to exclusion from work in healthcare institutions. In this study, 388 staff members in 42 nursing homes in Oslo County responded to questions about personal experience with MRSA and of own attitudes to challenges associated with the control and treatment of MRSA patients. Half (52%) of the nursing staff were concerned of becoming infected with MRSA and the consequences of this would be for own social life, family, economy, and work restriction. The concern was associated with risk factors like old buildings not suitable for modern infection control work, low staffing rate (70% without specific training in healthcare and 32% without formal healthcare education), defective cleaning and decolonization, and lack of formal routines and capacity for isolation of MRSA patients. Since the Norwegian MRSA guideline permits patients with persistent MRSA infections to move freely around in nursing homes, the anxiety of the staff to become infected and excluded from job was real.

## 1. Introduction

Nearly 1% of the Norwegian population live in nursing home with complex medical problems, high age (median 84 years), and need of assistance for daily living (>95%) [1–5]. Healthcare-associated infections (HAIs) are registered in 7% of these patients, 1% higher than in Norwegian hospitals, and the consequences are severe [6–8]. The Norwegian Infection Control Act demands written infection control programmes in nursing homes [5, 9, 10].

 In spite of international guidelines for infection prevention and control, nursing homes in Europe and USA may have large reservoirs of MRSA, affecting patients and staff, and resulting in persistent carriage [11–19]. Colonised cases may spread MRSA in the environment and via air [19–24]. The index person is often a staff member who may transmit MRSA via hands [21–24]. 

**Figure 1:**
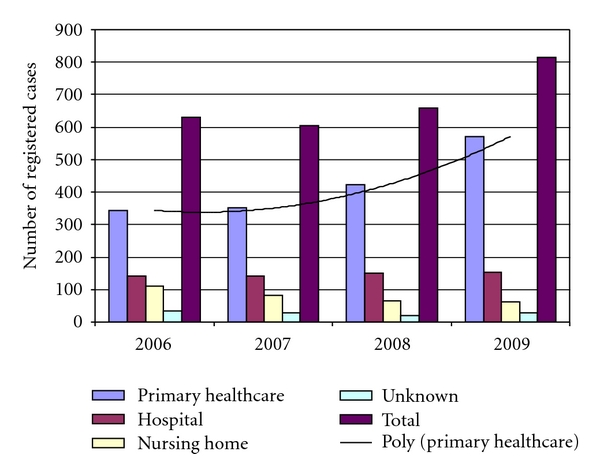
Development of MRSA (all cases) in Norway 2006–2009.

 MRSA is still increasing in Norway, especially in the primary health care, associated with imported cases and outbreaks in long-term care, affecting both patients and personnel [25–27] (Figure 1). In the Oslo County, 10% of all MRSA cases were healthcare personnel [26]. The outbreaks in nursing homes were associated with old buildings, insufficient isolation procedures and a less strict MRSA policy, failed eradication attempts, dement patients walking around, staff working part time at several institutions at the same time, overcrowding and mixture of patients, and lack of formal healthcare education [5, 10, 25–27].

 The Norwegian MRSA Control Guideline recommends testing of HCW exposed to MRSA [28]. Carriers are excluded from healthcare-related work until negative [28]. On the other hand, persistent carriers among the patients may move freely in the nursing home without any restrictions, and thereby expose personnel and other patients for MRSA [26–28]. The present study describes the staff's personal experience and attitudes to challenges in nursing homes, associated with the control and treatment of MRSA patients.

## 2. Materials and Methods

### 2.1. Nursing Homes

Forty two of 55 nursing homes in Oslo City participated, including 3350 beds with a mean of 102 beds per institution. They were divided into units of 10–25 patients, providing 24 hours care [5, 10]. Leaders and sisters worked daytime, while nursing personnel worked on three-divided shift. Only 19% of the staff were nurses, 70% were without specific training in healthcare (students), and 32% had no formal healthcare education at all.

### 2.2. The Staff Study and Questionnaire

Leaders and personnel were asked about infection control routines and knowledge and practice concerning MRSA [5]. The study was anonymous. This paper is based on data about infection control routines and the healthcare workers' personal feelings and attitude concerning challenges associated with the control and treatment of MRSA patients in nursing homes. The questions were about challenges like the quality of life of the MRSA-infected patient, the staffing situation, the extra need for cleaning, disinfection and decontamination, the functional standard of the buildings, the economic situation, and the need for information. 

 The nurse responsible for infection control distributed the questionnaire to the respondents [5]. In all, 528 questionnaires were delivered: 324 to nursing staff, 162 to sisters, and 42 to institution leaders. 

 The questions were ranged on a scale from 1 (no problem) to 5 (a very large problem).

*Statistical data* were analysed by SPSS 15.0 (SPSS Inc., Ill, USA).

## 3. Results

### 3.1. Respondents

Most nursing homes in Oslo (76.4%) participated, caring for approximately 3350 patients in 126 wards, with mean 26 beds in each. The rate of responding was 73.5% (388/528). The 388 respondents were 229 nursing staff (nurses and assistant nurses), 126 sisters, and 33 institutional managers [5]. The nursing staff was 90% women, mean age 41 years, and mean time in work position 7.4 years. All had a work position of 50% or more. 18% worked part time at several other institutions. The sisters were mostly women (87%), with mean age 46 years and mean time in work position 7 years. Among the 33 institution managers, 73% were women. The mean age was 50 years.

### 3.2. Nursing Home Standard

Most nursing homes were old buildings: the oldest from 1860 and the newest from 2001. Half (52%) of the buildings were estimated unsuitable for modern care of patients. The 3350 beds were in 2764 single rooms (83%) and 586 (17%) double rooms. Bathrooms were usually common for two or more residents. There was no isolation facility in the 42 institutions.

### 3.3. Earlier Experience with MRSA Cases

**Figure 2:**
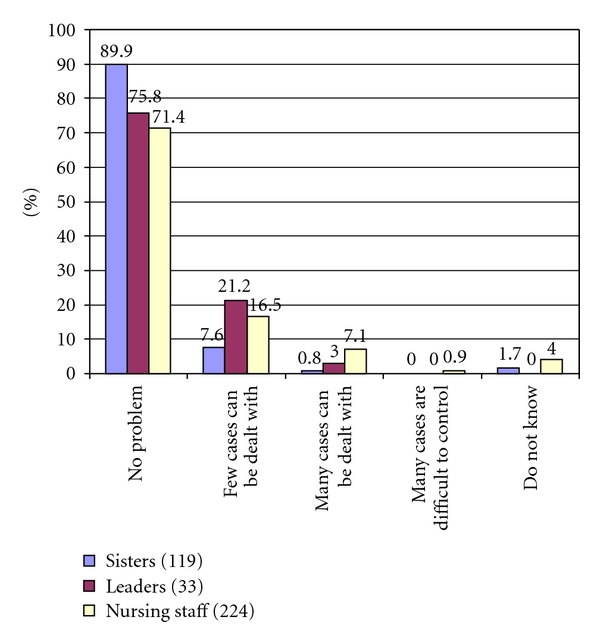
The size of the MRSA problem at your ward.

In all, 17% of the sisters had experienced at least one MRSA case at their unit during the last year. MRSA caused usually no problem (Figure 2). Only 7% of the nursing staff, 3% of the sisters, and 0.8% of the managers informed that they had many MRSA cases, but still the problem could be controlled.

### 3.4. The Staff's Concern of Becoming a Carrier

Half (52%) of the nursing staff was worried of being infected or colonised with MRSA and about consequences for their social life, family, and work situation (Table 1). One of the respondents answered: “the consequences of becoming a carrier of MRSA as a HCW could be serious for my private economy and work quarantine. This is the reason why I try to get a new job outside the healthcare system.” Six out of 18 sisters (33%) who had experienced MRSA on own unit were concerned about the problem of MRSA, compared to 50 out of 96 (52.1%) without this experience.

### 3.5. Challenges

**Figure 3:**
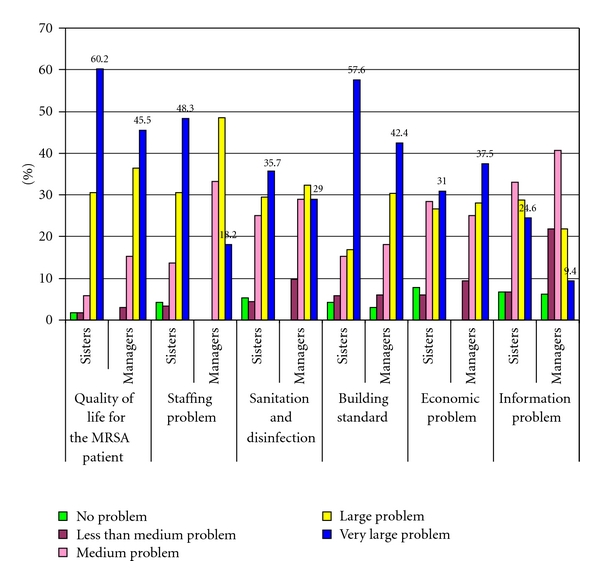
Nursing home problems associated with MRSA: quality of life, staffing, building standard, economy, information, sanitation, and disinfection.

The quality of life of an MRSA-infected patient was rated as a large challenge (Figure 3). The comments of the respondents were as follows: “It is a large ethical problem to isolate elderly people.”, “MRSA is mostly a serious problem for the infected person-himself”, and “The MRSA-infected resident should be treated medically and socially with contact and stimulation as entitled to.” 

 The staffing situation was estimated as a large challenge (Figure 3). The general staffing rate was low with 70% without specific training in healthcare and 32% without formal healthcare education.

 The increased need of cleaning, disinfection, and sanitation of the environment and of the MRSA-infected patient seemed also to be a problem for the staff. One respondent described the situation as follows: “Low staffing rate, many non-healthcare personnel during weekends, few cleaning personnel and other problems.” 

 The functional standard of the buildings was estimated as a large problem. A respondent commented the question: “The buildings are not constructed to take care of MRSA infection in the nursing home”. Another respondent commented as follows: “Nursing homes have no isolation facilities and we do not have bathrooms for each patient—so that is the largest challenge.” 

 The economic situation concerning outbreak of MRSA seemed to be a medium to large problem, especially for the managers. The information to personnel, patients, relatives, hospital, and others was more important for the sisters than for the institution managers.

### 3.6. Ranging the Challenges during Outbreaks of MRSA

The quality of life of a patient with MRSA was ranged as the largest challenge (Table 2). The sisters then ranged successively lack of staffing (more staff for isolation work, etc.), nursing home standard (lack of isolation facility, sharing rooms and bathrooms), and increased need for cleaning, disinfections, and sanitation, as large problems, while economy and information was a medium large problem. The institutional managers were mostly concerned about the nursing home standard, followed by staffing, economy, and sanitation.

## 4. Discussion

Close living proximity, use of antibiotics, and the presence of pressure sores and indwelling devices make nursing home residents ideal for spreading MRSA [29]. MRSA-colonised residents are up to six times more likely to develop infection than noncolonised ones and so increase their risk of dying [29]. 

 In this study, 388 staff members and managers in 42 nursing homes in Oslo County responded to questions about personal experience with MRSA and of attitudes to certain challenges associated with the control and treatment of MRSA in nursing homes. Problems were associated with old building standards not suitable for modern infection control work with lack of isolates, single rooms and bathrooms, a low rate of professional healthcare staffing and education in infection control work, and increased workload associated with cleaning, disinfection, and decolonizing. In addition, there was no isolation of persistent MRSA carriers, increasing the risk of transmission. 

 The weakness and limitation of the study was that the data were self-reported and could not be controlled by direct observation or additional questions [30]. Furthermore, our informants were selected, and personnel without health qualifications did not participate in this study. 

 Half of the nursing homes in Oslo County were not suitable for modern care of patients with no isolation facility in institutions caring for 3350 patients. Most patients shared bathrooms, and 20% shared rooms. Lack of isolates increases transmission of MRSA which may persist on several body sites, are transmitted by personnel's hands, and dispersed into the environment and by air within 24 hours [19–21, 23, 31, 32]. Single rooms with own bathroom should be the standard in nursing homes since sharing room increases the general risk of microbial transmission [33]. 

 Untrained and uneducated personnel working in nursing homes increases the risk of spread of MRSA [34]. This was the situation for one-third of our staff. Part-time work at different health institutions may also increase the transmission risk. MRSA control guidelines for exposed patients and personnel were often not implemented [5]. 

 The MRSA-infected patient was the largest challenge in nursing homes, since isolation may create an ethical problem. Therefore, infected residents should be decolonized at once and treated with contact and stimulation during the isolation period. The eradication method should be formalized and further studied to reach the best procedure for control. Still, some patients may become persistent carriers and should be taken especially care of [18, 25–27]. 

 However, persistent carriers are, according to the Norwegian MRSA guideline, allowed to move freely in the nursing home, exposing unprotected patients and personnel for MRSA [28]. Exposed personnel are recommended to be tested for MRSA and if they are infected, they are excluded from healthcare work, also in nursing homes until documented MRSA negative [28]. Thus, this inconsequent guideline may lead to increased spread of infection among patients and personnel in nursing homes, increased work restrictions, and increased fear of being infected. 

 Half of the nursing staff in our study worried about being excluded from work because of MRSA carriage and the consequences of this would be for own social life, family and economy. The “twenty-first century lepers” because of MRSA carriage is already a problem and may be an even larger problem among personnel in healthcare [35, 36].

## 5. Conclusion

Half of the staff in nursing homes in Oslo County worried of being infected by MRSA. The reason could be that half of the nursing homes were not built for modern patient care. A low staffing rate and high number of uneducated personnel increased the risk of transmission, as did problems with environmental hygiene and eradication of the MRSA status. In addition, persistent carriers moving freely in the nursing home would increase the transmission risk. 

 To control MRSA and other resistant microbes, it is mandatory to enhance building standards, including isolates and single rooms with bathrooms. Furthermore, prevention of MRSA is dependent on a good quality care by well-educated staff not working part time at several institutions. The infected patient should be decolonized and taken care of in the best ethical way, without risk for transmission to unprotected patients, visitors, and staff. In the future, MRSA infection should be defined as a “consumer empowered, rare, and unacceptable event,” also in nursing homes [37–40].

## Figures and Tables

**Table 1:** Reasons for being concerned about MRSA among nurses.

	Yes/	Yes
	answered	%
Afraid of getting an infection with MRSA	77/84	91
Afraid of becoming an MRSA carrier	100/101	99
Afraid of loosing my job because of MRSA carriage	45/59	76
Concerned about consequences for my social life if I become a carrier	65/70	93

**Table 2:** Ranging the challenges associated with MRSA in nursing homes.

Sisters		Institution managers	
Quality of life for the MRSA patient	90,7%	Quality of life for the MRSA patient	81,9%
Personal staffing	78,8%	Nursing home standard	72,7%
Nursing home standard	74,5%	Personal staffing	66,7%
Cleaning, disinfection, and sanitation	65,2%	Economy	65,6%
Economy	57,5%	Cleaning, disinfection, and sanitation	61,3%
Information	53,4%	Information	31,3%
